# Direct‐to‐Biology: Streamlining the Path From Chemistry to Biology in Drug Discovery

**DOI:** 10.1002/cmdc.202501080

**Published:** 2026-02-20

**Authors:** Ariane F. Hübner, Fabian Barthels

**Affiliations:** ^1^ Institute of Pharmaceutical and Biomedical Sciences (IPBS) Johannes Gutenberg‐University Mainz Germany

**Keywords:** combinatorial chemistry, direct‐to‐biology, drug discovery, high‐throughput screening, nanomole synthesis

## Abstract

Direct‐to‐biology (D2B) has emerged as a transformative concept in early drug discovery, defined by the direct on‐target screening of crude reaction mixtures without prior purification. First coined in 2021, the approach builds on advances in nanoscale synthesis platforms and was shaped by seminal studies that demonstrated the feasibility of plate‐based microscale chemistry for library generation. Today, D2B is increasingly adopted in academia and industry, with campaigns exploring diverse reaction classes, targeting modalities, and assay platforms. Thus, very recently, the first commercial providers now offer D2B services for ligand optimization, further driving adoption. Yet, despite clear advantages in speed, cost, and sustainability, D2B also faces limitations from assay interference and technical constraints in reaction miniaturization. Looking ahead, integration with AI‐driven design and high‐content biology promises to expand the scope of D2B and position it as a robust complement to traditional discovery paradigms.

## Graphical abstract

Crude yet clever: Direct‐to‐biology workflows exploit nanoscale synthesis and plate‐based assays to accelerate hit discovery, save precious intermediates, and rethink early drug discovery.

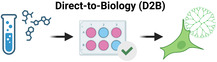

## 1 Introduction and Origin

Direct‐to‐biology (D2B) denotes workflows in which newly, in‐parallel synthesized compounds, often as crude reaction mixtures or minimally purified products, are tested directly in biological assays, collapsing the traditional make‐purify‐test cycle into rapid make‐test loops. The initial origin of the term is disputed, but it was popularized in a 2021 publication by Bush and coworkers through a GSK pharma‐led “D2B‐HTC” platform that generated >1000 molecules in 384‐well plates within 24 h and screened them without purification against drug targets, establishing practical feasibility and a vocabulary for the field [1]. Conceptually, D2B rests on advances in nanomole‐scale, high‐throughput wellplate‐based synthesis that matured over the 2010s, with publications introducing synthetic technologies for high‐throughput plate‐based synthesis, yet without a dedicated biological confirmation of synthesized molecules [2, 3, 4, 5, 6, 7]. From roughly 2014 to the term's definition of “Direct‐to‐Biology,” the groundwork for a more integrated synthesis‐to‐screening paradigm was quietly laid by a series of incremental but conceptually aligned studies:

In 2014, an off‐rate screening (ORS) by surface plasmon resonance (SPR) introduced a kinetic hit‐to‐lead sampling directly from unpurified reaction mixtures, foreshadowing the idea of extracting biologically relevant information from crude products [8]. That same year, the activity‐directed synthesis (ADS) concept was introduced by Nelson et al., wherein reaction arrays were screened for function, and only the most promising crude mixtures were scaled up and purified, demonstrating a bioactivity‐guided synthesis loop [9, 10, 11, 12, 13, 14, 15]. In the following years, several publications with similar concepts were published [16, 17, 18, 19, 20, 21, 22]. This trend crystallized in 2018 with Cernak and colleagues’ nature study coupling nanomole‐scale reactions to affinity‐selection bioassays, thereby ranking drug potency without time‐intensive purification [23]. This early‐stage concept (first dubbed by Cernak as “nanoSAR” and later as “D2B”) demonstrated that chemical and biological readouts can be tightly integrated at a reaction scale, a core D2B tenet [23]. Yet, D2B is distinct from other nanoscale high‐throughput library generation methods such as DNA‐encoded libraries [24], bead‐based and split‐and‐pool‐derived immobilized libraries [25], or prefabricated peptide/nucleic‐acid arrays [26]: whereas these other approaches typically decouple synthesis from screening, for example, by solid‐phase or nucleic‐acid immobilization, D2B emphasizes on‐the‐fly synthesis in plate‐based layouts with immediate biological readout, enabling fast, iterative design‐make‐test cycles with minimal material and maximal learning per experiment.

## 2 Current Landscape of Direct‐to‐Biology Research

**FIGURE 1:**
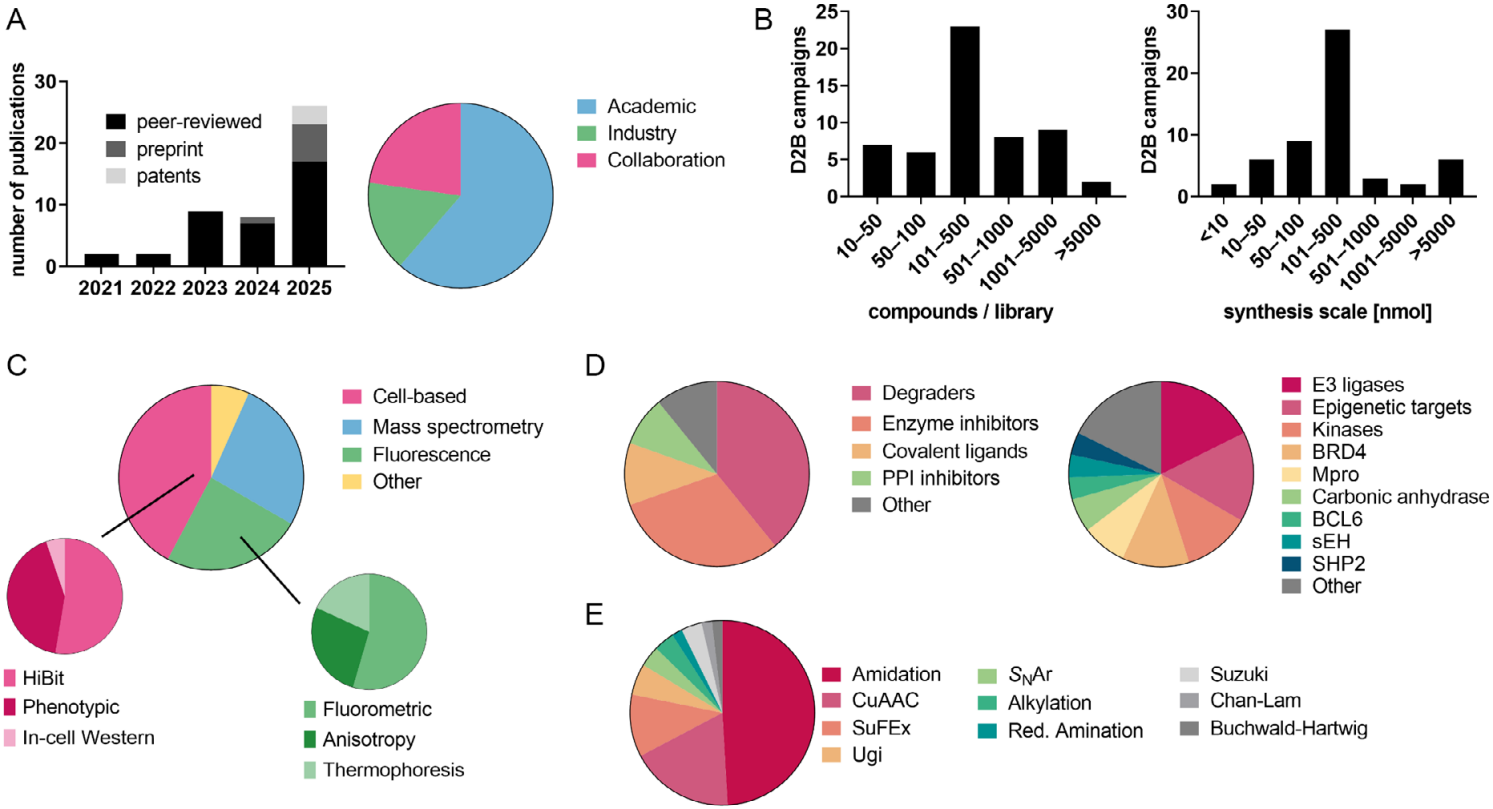
Current landscape and analysis of the direct‐to‐biology (D2B) research. (A) D2B publication format dynamics and authorship proportions. (B) Library size and reaction scales of published D2B campaigns. (C) Assay modalities for D2B biological evaluation per screening campaign. (D) Target landscape and modalities per screening campaign. (E) Reaction chemistry per D2B campaign.

In this review article, we summarize research publications that use the terms “D2B” or “nanoSAR” in the title or main body, published through January 2026. Studies were included if they reported plate‐based or nanoscale synthesis followed by direct biological evaluation of crude or minimally processed reaction mixtures, without chromatographic or other multistep purification. We analyzed D2B publications, encompassing both peer‐reviewed articles, patents, and preprints describing in situ synthesis campaigns coupled to immediate biological evaluation (*N* = 44). Preprints that later appeared as peer‐reviewed publications were consolidated into a single entry. Publications were annotated across multiple categories and dimensions; hence, a medicinal chemistry‐focused analysis of the D2B landscape is depicted in Figure 1.

### 2.1 Publication Dynamics and Authorship

Annual output increased from the first denoted D2B publication in 2021 to ~25 publications/year in 2025. The inflection in 2023/2024 coincides with the very recent adoption of nanoscale synthesis and campaign‐style optimization. Industry submissions and mixed academic‐industry collaborations account for ~40% of D2B campaigns, highlighting that both academia and the pharmaceutical industries are interested in this concept. Yet, so far, no significant differences regarding time‐to‐data metrics and methodological diversity could be noticed to distinguish academic from industrial D2B campaigns.

### 2.2 Library Sizes and Reaction Scales

Current D2B campaigns span library sizes from min. 50 to >5000 compounds (median 100–500 compounds/library). Reaction scales cluster at the triple‐digit nanomole scale for most campaigns but, depending on the application, can be as low as 1 nmol or as high as 10 µmol. Notably, campaigns with a library size >500 predominantly operate at ≤500 nmol per reaction, underscoring the coupling between throughput and material economy applicable for 96‐ or 384‐well‐based synthesis layouts.

### 2.3 Reaction Chemistry

The dominant D2B transformations are currently amide formations (50% of campaigns), copper(I)‐catalyzed azide–alkyne cycloaddition (CuAAC, 20%), sulfur(VI)fluoride exchange (SuFEx, 12%), and other late‐stage diversification chemistries followed by diverse arylation, alkylation, amination, and C—C cross‐coupling reactions. Notably, this aligns well with the frequency of reaction types used in conventional medicinal chemistry structure–activity relationship (SAR) studies but still diverges from the frequency of preferred chemotypes found in approved drugs [27, 28].

In general, selection of chemistry reflects the need for (i) biocompatibility with downstream assay technologies, (ii) robustness at the nanomole scale, mostly in well plates, and (iii) clean conversion profiles suitable for testing minimally purified mixtures. Over the past 5 years, the field has observed a slight shift toward click‐like transformations and an uptick in C—N/C—C cross‐couplings enabled by improved microscale synthesis protocols.

### 2.4 Assay Modalities

Cell‐free assays remain prevalent, with fluorescence‐based readouts (25%) and mass spectrometry (30%) dominating early triage; cell‐based assays constitute 40% and are increasingly used for proteolysis targeting chimera (PROTAC) screening or phenotypic profiling. In more detail, fluorescence‐based assays use either fluorometric, anisotropic, or thermophoretic readouts. Meanwhile, cell‐based assays mostly characterize biological effects using phenotypic or HiBiT screening technologies. Among other assays (5%), luminescence and high‐throughput crystallographic screening represent two rarely used assay modalities coupled with in situ D2B synthesis.

### 2.5 Target Landscape and Modalities

Classical inhibitor campaigns predominate in absolute number (60%), while reversible small‐molecule ligands only constitute half of those campaigns, and covalent and protein–protein‐interface ligands form the other half. Targeted degrader‐oriented campaigns (molecular glues (MGs) and PROTACs) constitute 40% of D2B publications and typically use convergent assembly (e.g., linker diversification, also known as “linkerology”) under nanoscale conditions to explore E3/warhead/linker space efficiently. Beyond E3 ligases and specifically BRD4‐targeting PROTACs, kinases and epigenetic enzymes account for 14% and 12% of addressed targets, often expanding fragment‐like D2B libraries.

### 2.6 Emerging Picture

The field is transitioning from demonstration‐scale experiments to platformized, iterative D2B workflows: small, robust reaction sets at nanomole scale; rapid, mostly purification‐sparing analytics; and assay choices that tolerate residual matrix. Recently, several commercial providers, including Concept Life Sciences, Domainex, WuXi AppTec, and Molecule.one, have begun to offer D2B‐based synthesis services specifically for the commercial optimization of existing ligands, further underlining translational traction and the potential for broader adoption in pharmaceutical and academic pipelines [29, 30, 31, 32]. Together, these trends point to D2B as a complement to conventional library‐based discovery, optimized for fast learning cycles per unit material.

## 3 Applications of Direct‐to‐Biology: Target‐Specific Insights

### 3.1 Kinase Inhibitors

Kinases are among the most prominent and well‐characterized target families in medicinal chemistry, due to their central role in cellular signaling and druggability for cancer‐related diseases, and hence, their biological relevance makes them also attractive targets for D2B strategies [33, 34]. Several D2B campaigns have been applied to kinase targets, including PROTAC‐based approaches (detailed in chapter 3.3.1, PROTACs”) as well as compound libraries derived from known inhibitors of mitogen‐activated protein kinase kinase 7 (MKK7) and cyclin‐dependent kinase 2/cyclin E (Cdk2/CycE) [35, 36, 37, 38, 39, 40].

**FIGURE 2:**
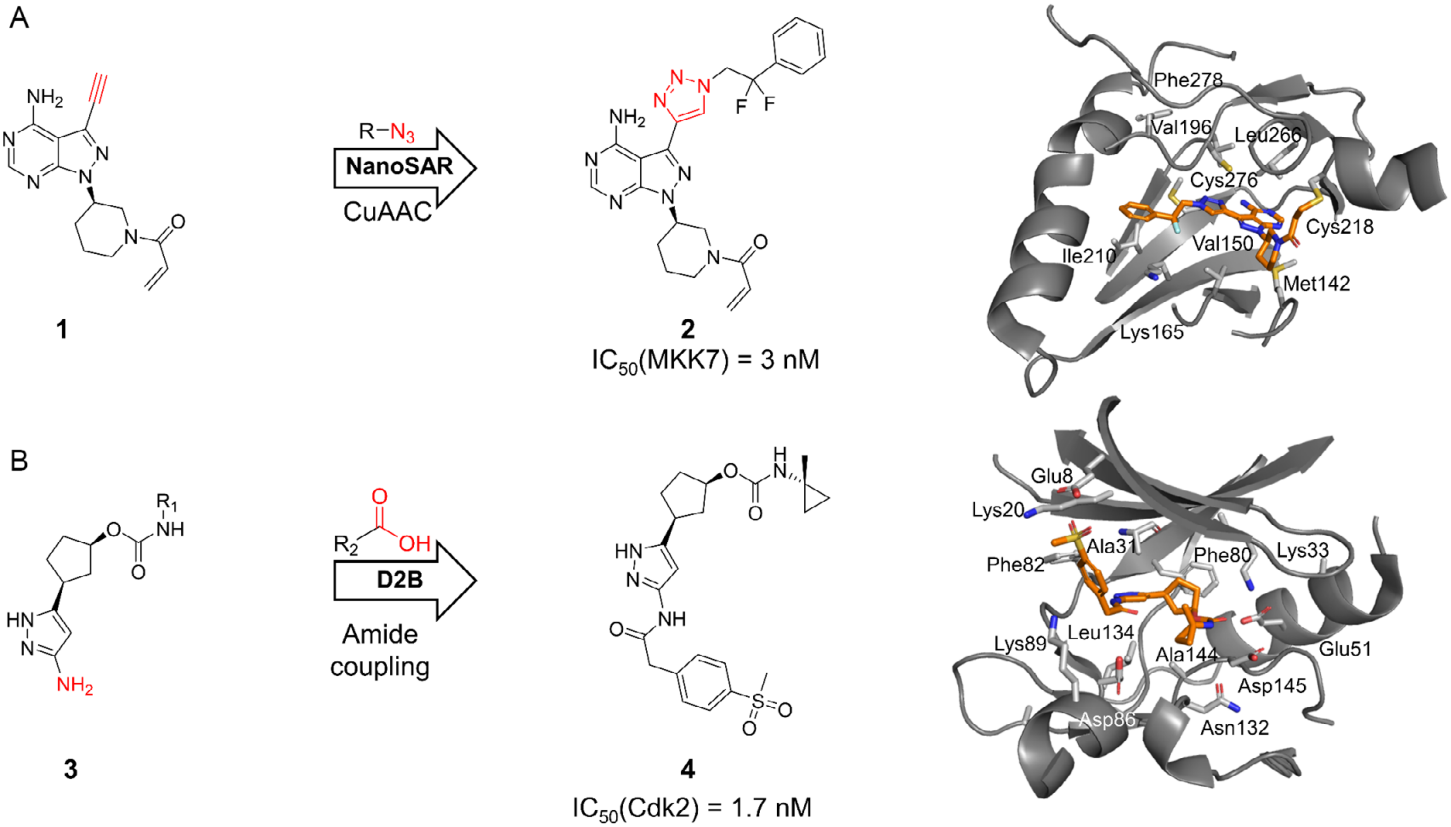
D2B campaigns targeting kinases. (A) Optimization scheme and X‐ray crystal structure (PDB: 7OVJ) of the CuAAC D2B campaign reported by Gehrtz et al. targeting MKK7 [35]. (B) D2B campaign conducted by the Cernak group targeting CDK2 via amide diversification, resulting in compound **4** (X‐ray crystal structure PDB: 9OB2) [36].

In one of the first influential studies, Gehrtz et al. used a covalent ibrutinib derivative (**1**), bearing an acrylamide warhead and alkyne handle, to generate inhibitor analogs via copper(I)‐catalyzed azide–alkyne cycloaddition (CuAAC) to target MKK7 (Figure 2A) [35]. Using a droplet‐ejection system, they coupled 448 commercially available azides in a 384‐well format and screened the crude mixtures directly using an in‐cell Western assay. CuAAC is particularly suitable for D2B efforts due to its high efficiency, aqueous compatibility, biocompatibility, nontoxic reagents, and the broad commercial availability of azide libraries [41, 42]. This MKK7‐targeting strategy yielded potent inhibitors with IC_50_ values of 3–4 nM (compound 2) and EC_50_ values of 265–380 nM, which displayed major improvements compared to the parent compound 1 (EC_50_ > 10 µM) [35]. Furthermore, cocrystal structures with MKK7 were elucidated, providing insights into their binding modes (Figure 2A).

During a second kinase‐targeting D2B study, Douthwaite et al. applied this concept to explore Cdk2/CycE inhibitors (Figure 2B) [36]. Through a high‐throughput combinatorial screen, they coupled six aminopyrazole cores (compound **3** among others, Figure 2B) with 128 carboxylic acids to generate a 768‐membered library. To maximize reaction efficiency, they systematically combined different bases (DIPEA, NMI) with coupling reagents (CDI, HATU), achieving a synthetic success rate of 90% using an automated liquid handling system. The crude reactions were tested with ADP‐Glo and off‐chip Caliper mobility‐shift assay and subsequently were tested orthogonally using an affinity selection‐mass spectrometry assay (ASMS). Notably, during this study, ligand‐containing crystal structures could also be obtained directly from crude reaction mixtures (compound **4** in Figure 2B).

### 3.2 SARS‐CoV‐2 Main Protease (M^Pro^) Inhibitors

The COVID‐19 pandemic has sparked intense interest in antiviral drug development, particularly targeting the main protease (M^Pro^) of SARS‐CoV‐2, which plays an essential role in viral replication [43, 44]. Accordingly, multiple D2B approaches have been employed to explore this enzyme as a therapeutic target, while notably, several of these D2B campaigns have employed nanoscale synthesis, which relied on amide coupling reactions to generate libraries of diverse amide derivatives [45, 46, 47, 48].

**FIGURE 3:**
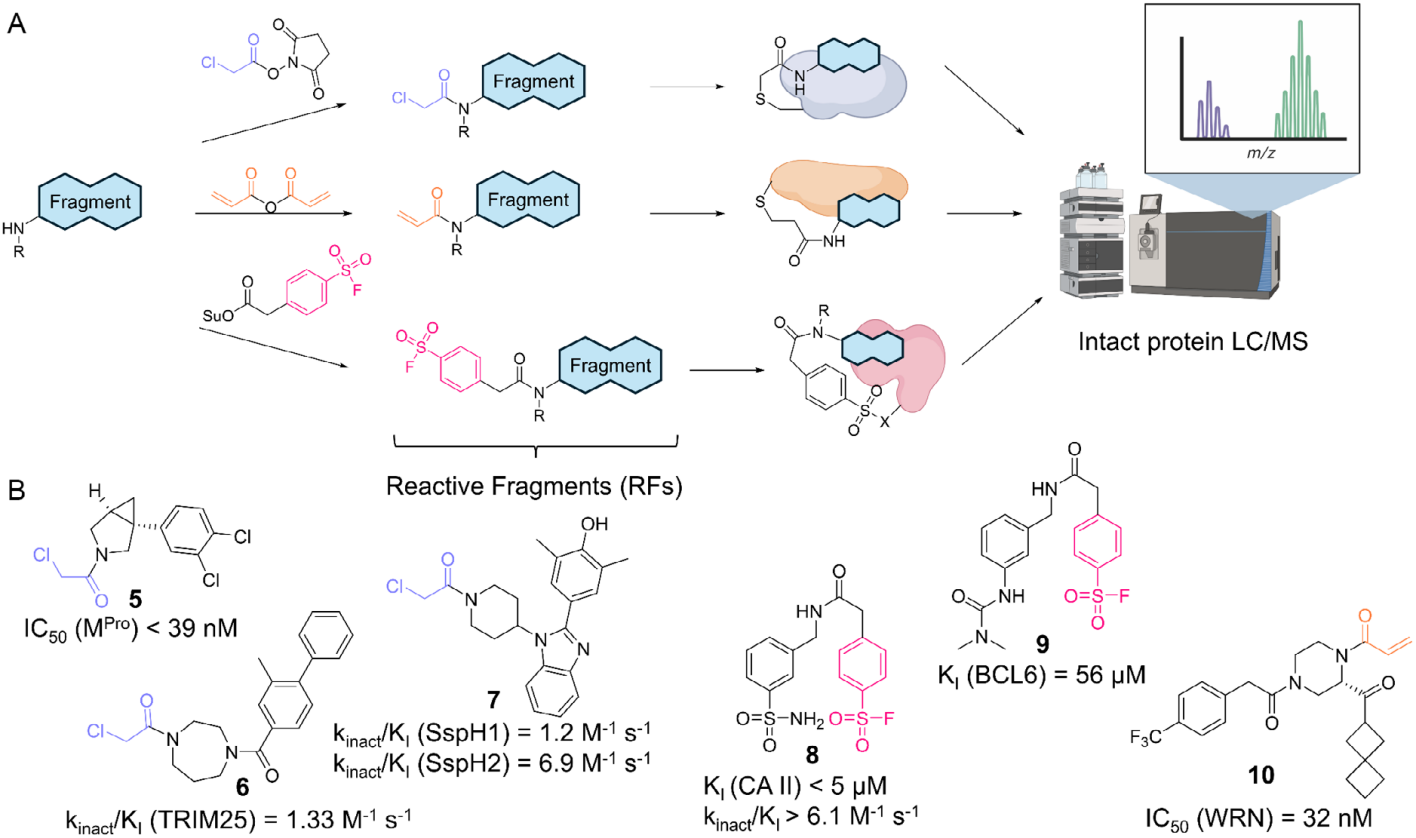
Reactive fragment (RFs) screening strategy introduced by Bush and coworkers. (A) Scheme illustrating the workflow of different RF‐D2B strategies (chloroacetamides, acrylamides, and sulfur(VI) fluorides). Fragments containing amine groups are reacted with a covalent warhead transfer reagent to form a nanoscale library of the corresponding RF, which is incubated with the target enzyme and analyzed by intact protein LC/MS. (B) Covalent D2B inhibitors identified by multiple RF‐D2B screenings on different target enzymes [48, 49, 50, 51, 52].

**FIGURE 4:**
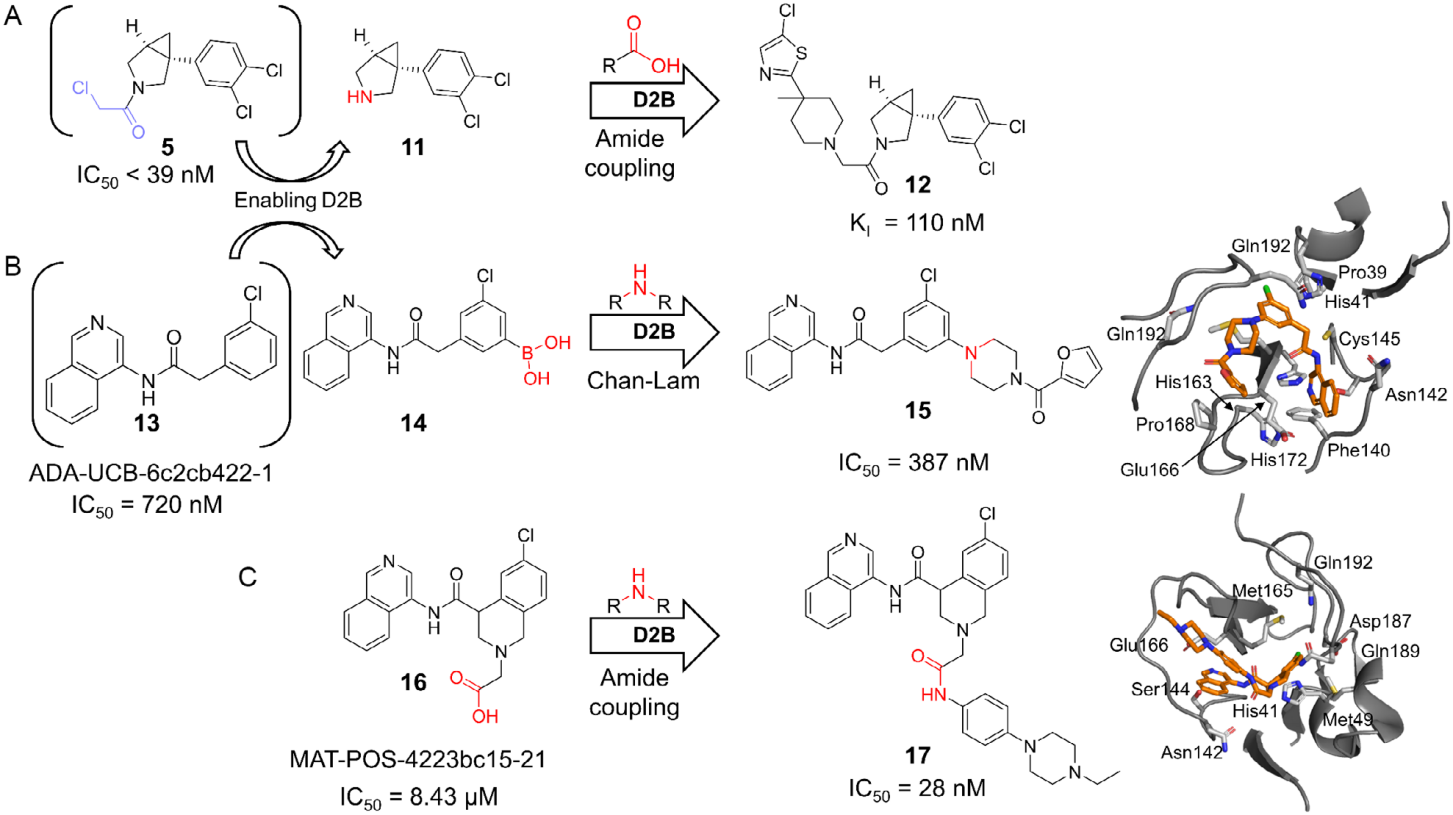
D2B campaigns targeting the SARS‐CoV‐2M^Pro^. (A) Scheme illustrating the transformation of reactive fragment **5** into a noncovalent M^Pro^ inhibitor **12** by D2B amidation [48]. (B,C) D2B campaigns reported by the Moonshot open‐science initiative, featuring Chan–Lam and amidation D2B to generate M^Pro^ inhibitors **15** and **17** (X‐ray structures PDB: 7GJZ, 7GNL) [46].

Recently, Bush and coworkers have applied a distinct D2B strategy starting from cysteine‐targeting chloroacetamide reactive fragments (RFs) to identify covalent (Figure 3) and noncovalent (Figure 4) M^Pro^ inhibitors [48]. An initial screen of 219 purified RFs yielded a covalent hit, which was elaborated via a D2B library of 193 amines reacted with the N‐hydroxysuccinimide ester of chloroacetic acid to form chloroacetamide‐decorated inhibitors. Screening of these inhibitor candidates by intact protein LC/MS identified M^Pro^‐targeting compound **5** with an IC_50_ smaller than 39 nM (Figure 3B). It is noteworthy that, beyond M^Pro^, this covalent inhibitor‐focused D2B screening strategy could be harnessed for multiple other targets (including TRIM25, SspH1, BCL6, and WRN) and diverse electrophilic warheads (including chloroacetamides, acrylamides, and sulfonyl fluorides) [48, 49, 50, 51, 52]. The most potent covalent D2B inhibitors (**5**–**10**) are summarized in Figure 3B and described in detail in the respective chapters 3.3–3.7.

Subsequently, to discover noncovalent M^Pro^ inhibitors, a cascade D2B campaign was performed in which the covalent warhead of the initial hit **5** was replaced, followed by HATU‐mediated amide coupling with two iterative carboxylic acid libraries (first 146 diverse building blocks and subsequently 72 focused candidates), ultimately yielding noncovalent inhibitor **12** with a *K*_I_ of 110 nM (Figure 4A) [48]. In this context, another amidation‐focused D2B study of the Cernak group started from a previous in silico screening campaign and, through RapidFire MS‐based screening, identified a hit compound with an IC_50_ of 5.06 µM [45].

Two additional M^Pro^‐targeting publications, including the open‐science COVID Moonshot initiative and a study led by the King‐Smith group, were also utilizing amidation‐stirred D2B optimization of M^Pro^ inhibitors [46, 47]. These amidation‐stirred campaigns yielded compounds with binding affinities in the low nanomolar range, targeting the P3/P5 pocket of M^Pro^ (e.g., compound **17**, IC_50_ = 28 nM) [46, 47]. The COVID Moonshot project not only employed amide coupling but also pioneered another chemically distinct D2B strategy, uniquely applying a Chan–Lam to compound **14** derived from a fragment‐based crystallographic screen (compound **13**) (Figure 4B,C) [46]. This led to compounds targeting the P4 pocket of M^Pro^, with the most potent compound, **15,** exhibiting an IC_50_ of 387 nM (Figure 4B). It is worth noting that the amide coupling reactions were overall more successful than the Chan–Lam reactions, with 151 out of 300 reactions yielding >30% product compared to only 29 out of 300 for the Chan–Lam transformations.

### 3.3 Epigenetic/Epitranscriptomic Inhibitors

Epigenetic and epitranscriptomic targets have become increasingly relevant due to their key roles in gene regulation and disease progression. Consequently, D2B strategies have enabled the exploitation of the unique features of the epigenetic landscape for accelerated drug discovery [53, 54].

**FIGURE 5:**
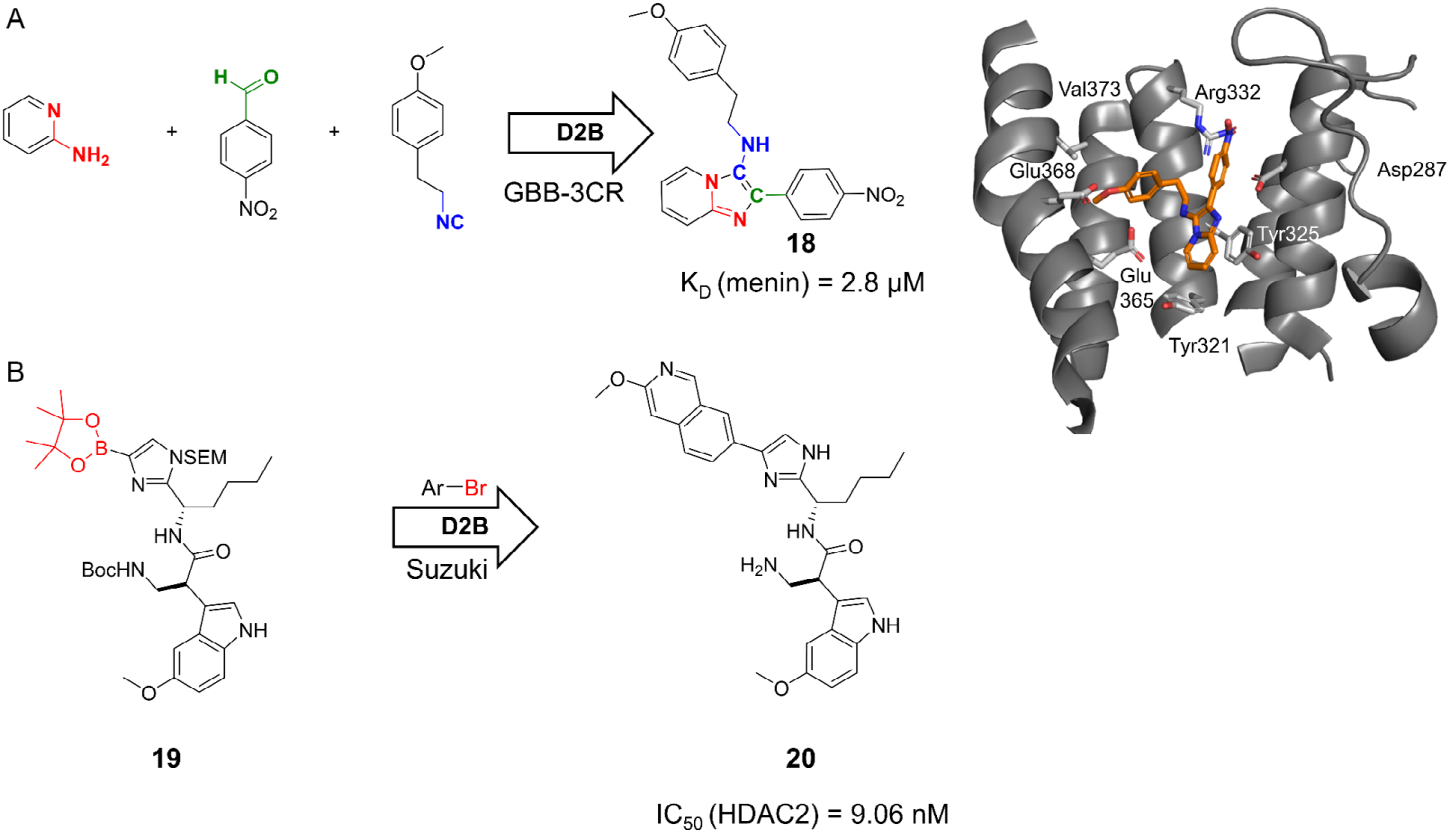
D2B campaigns targeting selected epigenetic drug targets. (A) GBB‐3CR‐based D2B strategy utilizing 38 amidines/aminopyridines, 53 aldehydes, and 71 isocyanides, resulting in menin inhibitor **18** (X‐ray structure PDB: 6S2K) [57]. (B) Suzuki coupling‐based D2B campaign yielding HDAC2 inhibitor **20** [58].

In the context of D2B, multicomponent reactions (MCRs) provide efficient access to large, diverse libraries with drug‐like properties and hence, provide an opportunity for large combinatorial screens [55, 56]. In one of the first conceptual D2B publications, Dömling and colleagues applied the Groebcke–Blackburn–Bienaymé reaction (GBB‐3CR) to generate 1536 compounds (from a theoretical space of 142,994) and screened crude products via differential scanning fluorimetry assays (DSF) to identify Menin‐MLL inhibitors (Figure 5A) [57]. From this screening, 22 hits were resynthesized and validated by microscale thermophoresis (MST) assay, with the most potent compound, **18,** showing a *K*_D_ of 2.8 μM.

In a complementary study targeting histone deacetylase 2 (HDAC2), a 70‐membered compound library was synthesized via amide coupling and Pd‐catalyzed Suzuki–Miyaura reactions on literature‐reported scaffolds of HDAC inhibitors (Figure 5B). The crude reactions were screened by the Automated Ligand Identification System (ALIS) and microsomal stability assays, which revealed that amide‐derived compounds showed better correlation between ACE_50_ and biochemical‐assessed IC_50_ values than Suzuki products [58]. During this study, lipophilic ligand efficiency (LLE) and Lipophilic Metabolism Efficiency (LipMetE) calculations also supported the selection of D2B candidates with a favorable balance of potency, lipophilicity, and metabolic stability for future drug development. The most potent inhibitor, **20,** with an IC_50_‐value of 9.06 nM, is shown in Figure 5B.

Beyond traditional activity‐based screening, advanced D2B platforms have recently combined automated synthesis and structural biology. One such campaign integrated the usage of liquid handlers for synthesis coupled with high‐throughput protein crystallography to evaluate 1696 fragment‐extended compounds targeting pleckstrin homology domain‐interacting protein (PHIP2), yielding 969 diffraction datasets and 29 cocrystal structures [59]. Although the initial fragment showed no activity in solution‐based assays, D2B optimization led to a compound with *K*_D_ and IC_50_ values of 50.03 and 31.15 μM, respectively. Subsequently, Biggin and colleagues applied the results from this crystallography study in “xSAR” calculations to further evaluate the screening results from this study (see chapter 4, “Computational D2B Technologies”) [60].

To support high‐throughput screenings (HTS) and D2B approaches of other epigenetic and epitranscriptomic targets, fluorescent methyltransferase tracers were developed via CuAAC in a D2B workflow and tested via MST and fluorescence polarization (FP) assays [61]. This led to the identification of novel displacement tracers for multiple epitranscriptomic drug targets, including METTL3/14, METTL1/WDR4, and bacterial methyltransferases.

### 3.4 Targeted Degraders

D2B has been employed for several discovery campaigns of targeted degraders. In this review, we differentiate between divalent PROTACs (chapter 3.4.1) and monovalent MGs (chapter 3.4.2). Due to the synthetic complexity of heterobifunctional degraders and the limited predictive power for the degrader development, SAR exploration remains largely empirical and resource‐intensive [62, 63]. In addition to nearly 20 peer‐reviewed publications that use D2B for degrader development, three patents employing the D2B technology for degrader development were published in 2025 [64, 65, 66].

#### 3.4.1 Proteolysis Targeting Chimeras (PROTACs)

D2B approaches are particularly well‐suited for PROTAC discovery, where hit identification typically requires high synthetic effort and offers limited opportunities for rational design, and thus, degrader development is the single most frequent application of recent D2B publications (Figure 1D). A key distinction from classical D2B enzyme inhibitor screening is that unreacted precursors during PROTAC development often retain target or E3 ligase‐binding activity, potentially contributing to cytotoxicity and influencing degradation readouts. Accordingly, many D2B‐PROTAC studies evaluate educts in both cytotoxicity and degradation assays, sometimes by mimicking incomplete conjugation [39, 40, 67]. Most PROTAC‐D2B campaigns employ HiBiT assays followed by mechanistic validation, such as Western blotting or inhibition of the proteasome or recruited ligase [37, 38, 39, 40, 67, 68, 69, 70, 71, 72].

**FIGURE 6:**
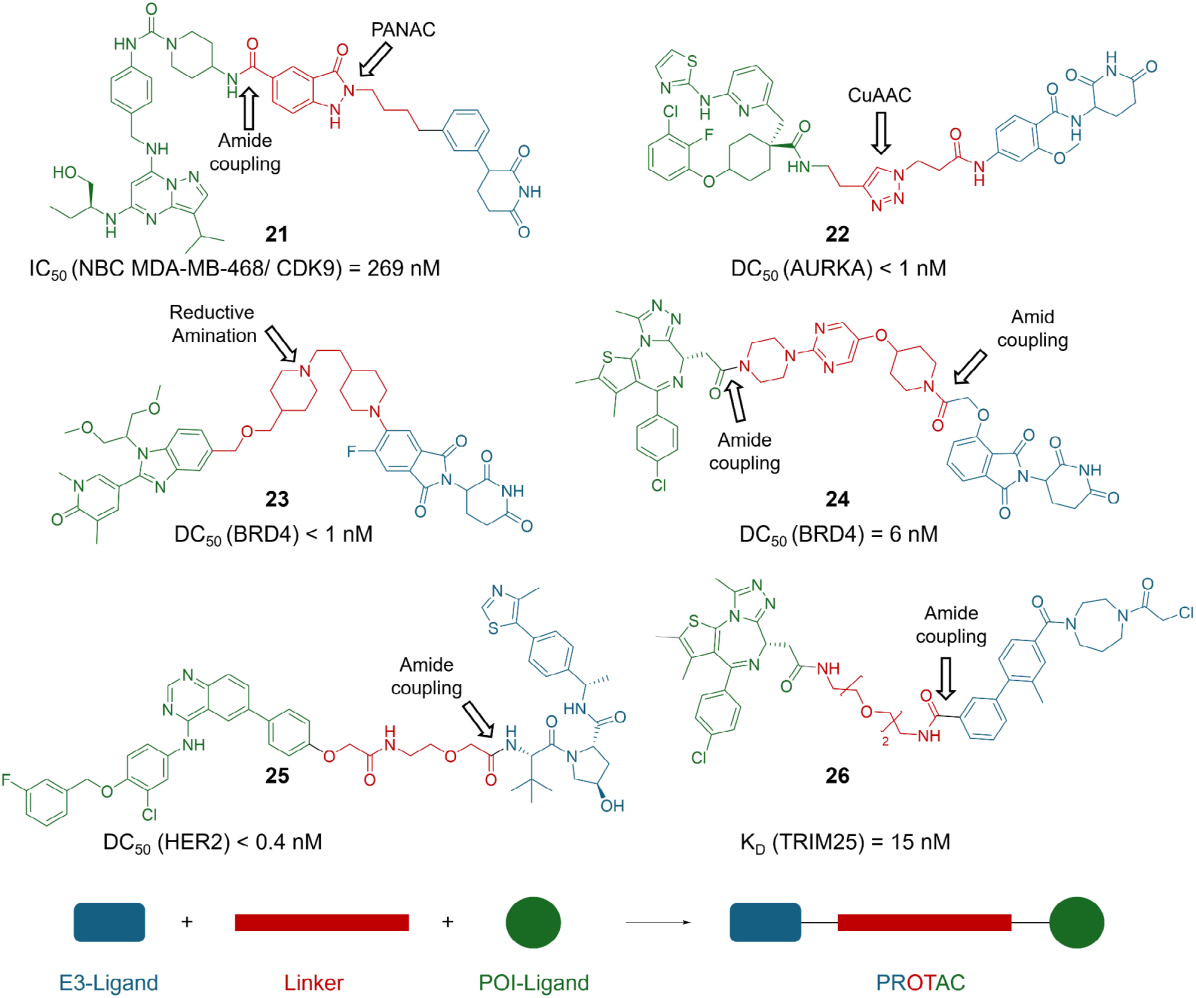
Overview of selected PROTACs synthesized via various D2B strategies [37, 39, 40, 49, 68, 69]. The recruiting chemotype for the protein of interest (POI) is highlighted in green, the linker in red, and the E3 ligase‐binder in blue.

In the current landscape of D2B‐PROTACs, cereblon (CRBN)‐recruiting PROTACs have often outperformed von Hippel–Lindau (VHL) ligands [37, 39, 69], while several campaigns have employed amidation strategies, for example, for bromodomain‐containing protein 4 (BRD4) targeting PROTACs using mono‐*N*‐Boc‐protected diamines in a coupling‐deprotection‐coupling workflow for modular assembly (e.g., compound **24**, Figure 6) [68]. Similar to many conventional PROTAC development studies, BRD4 remains a common proof‐of‐concept system for D2B‐PROTAC platforms, similar to non‐D2B degrader establishment [39, 40, 49, 67, 68, 69, 73]. For example, JQ1‐ or I‐BET469‐based libraries were synthesized via EDC‐ or HATU‐mediated coupling with diverse linkers and ligase ligands [67, 69]. In one case, three I‐BET469 acid exit vectors were screened and further optimized, yielding picomolar PROTACs (DC_50_ < 0.1 nM) [69]. Similarly, human epidermal growth factor receptor 2 (HER2)‐directed PROTACs based on lapatinib were synthesized using linker prefunctionalized E3 ligase ligands (Figure 6, compound **25**) [69].

Beyond CRBN and VHL‐based E3 ligases, Bush and colleagues employed a fragment‐based D2B approach (Figure 3, compound **6**) to identify novel covalent ligands for the E3 ligase tripartite motif containing 25 (TRIM25), in a manner analogous to previous efforts targeting M^Pro^ (chapter 3.2) [49]. In this study, TRIM25 binding fragments were linked via NHS esters to chloroacetamides warheads, guided by crystallography data to define the exit vector. Hence, the resulting JQ1‐based BRD4 PROTAC **26** of this new type of E3 degrader demonstrated SPR affinity in the nanomolar range (Figure 6) [49]. In a different D2B‐PROTAC strategy, amide coupling was combined with “PANAC” (primary amines and *o*‐nitrobenzyl alcohols cyclization) linker chemistry, utilizing amines and bifunctional linkers bearing NHS esters and *o*‐nitrobenzyl alcohols [37]. The “PANAC” reaction enables the intramolecular light‐induced cyclization of primary amines with *o*‐nitrobenzyl alcohols, forming heterocyclic scaffolds via a cyclization followed by dehydration and tautomerization [74]. This modular approach enabled the generation of 580 PROTACs targeting CDK9 in MDA‐MB cells, yielding the most potent CDK9 PROTAC, **21,** with an IC_50_ of 269 nM (Figure 6).

Also, CuAAC chemistry was recently used to generate PROTAC libraries targeting BRD4, Aurora kinase A (AURKA), soluble epoxide hydrolase (sEH), and WD repeat domain 5 (WDR5) inhibitors functionalized with solvent‐exposed alkyne‐functionalized target recruiters [39]. The subsequent reaction with 48 ligase‐binding azides (36 for CRBN, 12 for VHL) yielded 192 PROTACs per target with up to 92% conversion and subnanomolar DC_50_ values for AURKA (compound **22**, Figure 6). In another D2B study, two GSK3 ligands and 7 CRBN binding ligands were similarly linked to 12 CuAAC and S_N_2‐compatible linkers [38]. At AstraZeneca, D2B researchers explored the development of PROTACs targeting 2′‐deoxynucleoside 5′‐monophosphate N‐glycosidase (DNPH1) [72]. For this, two D2B synthetic approaches were pursued, each based on a distinct parent compound. The first (with a triazol core), derived from a DNA‐encoded chemical library (DECL) hit against DNPH1, was chemically modified to enable amide coupling with various E3 ligase recruiters via different linkers and an amine handle, affording a PROTAC with a DC_50_ of 405 nM [72]. The second (with a quinazoline core), originating from a prior HTS campaign, was functionalized with an aldehyde moiety to enable reductive amination with the same E3 recruiter library, yielding a highly potent PROTAC (DC_50_ = 28 nM, DC_max_ = 100%) [72].

Beyond CuAAC and amid coupling, other D2B‐compatible chemistries have occasionally also been explored: A solid‐phase Ugi reaction was employed to generate photocleavable BRD4‐targeting PROTACs, using linkers of varying length, composition, and rigidity, as well as CRBN ligands with diverse substituents [70]. Furthermore, in another proof‐of‐concept study, several reaction classes were adapted for D2B assembly of BRD4 PROTACs based on I‐BET469 [40]. In this regard, reductive amination with picoline borane delivered picomolar degraders, for example, compound **23** (Figure 6), while a Suzuki–Miyaura cross‐coupling was integrated into a three‐step sequence (cross‐coupling, Boc deprotection, and amidation). Finally, different alkylation strategies were demonstrated for RIPK2‐directed PROTACs, yielding both N‐ and O‐linked variants and further validating this method for D2B synthesis [40].

#### 3.4.2 Molecular Glues

**FIGURE 7:**
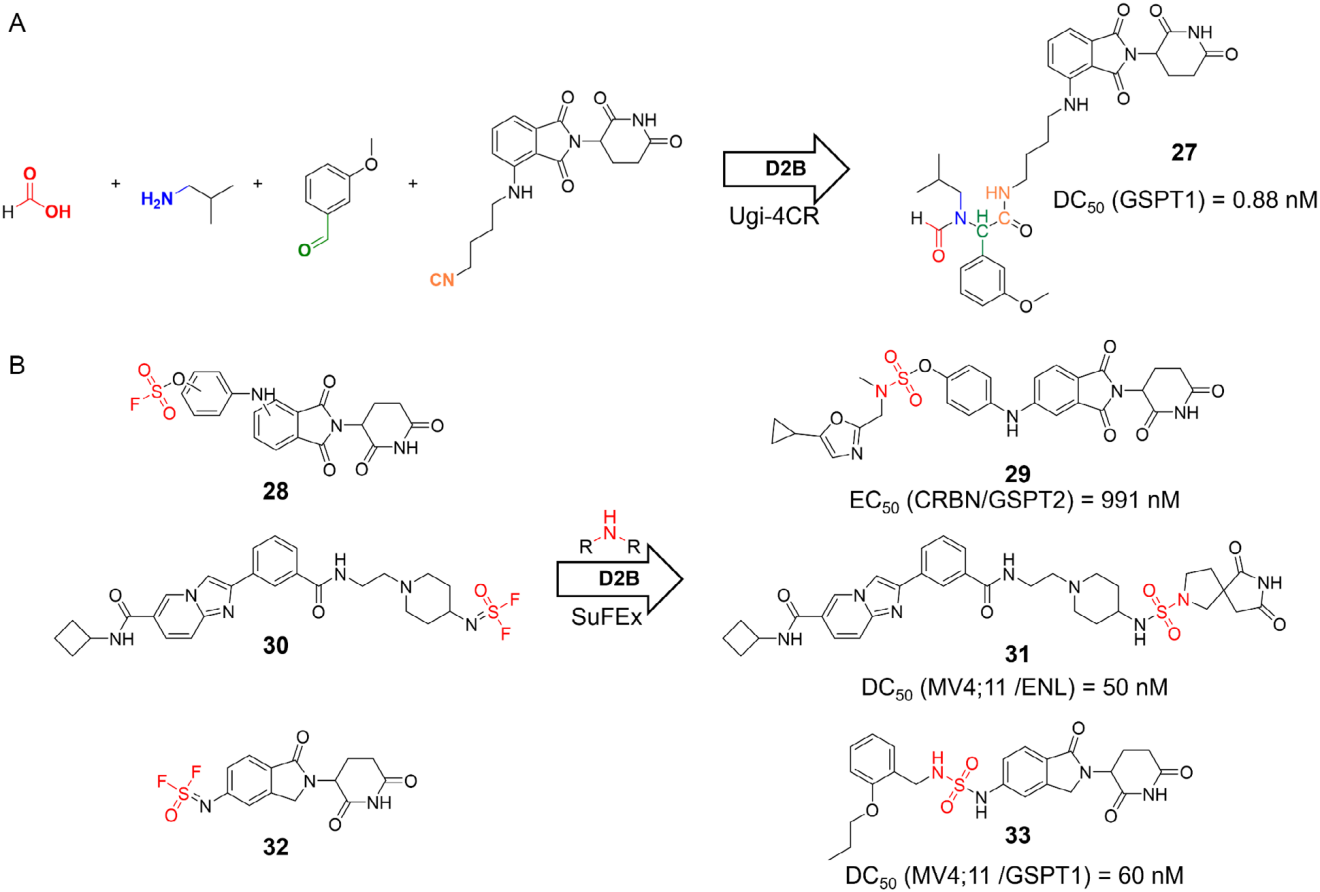
D2B campaigns for the development of molecular glue degraders. (A) Ugi‐4CR reaction‐stirred D2B campaign for the development of GSPT1 degrader **27** [77]. (B) SuFEx chemistry was employed in three different D2B campaigns for the discovery of molecular glues **29** **–** **33** [73, 75, 78].

Similar to PROTACs, MGs are often discovered serendipitously, and rational design remains highly challenging due to the lack of clear SARs [75, 76, 77, 78]. Consequently, large‐scale empirical screening remains a central strategy. Hence, SuFEx chemistry has gained traction in MG discovery owing to its robust reactivity and high conversion rates, especially in reactions of aryl fluorosulfates with primary and secondary amines (Figure 7B) [73, 75, 78]. Several MG campaigns have focused on CRBN‐recruiting scaffolds, commonly derivatizing CRBN ligands and screening for degradation phenotypes [70, 75, 76, 77, 78].

For instance, G1 to S phase transition 1 (GSPT1) degraders were identified via a Ugi‐based D2B screen using a pomalidomide‐derived isocyanide (Figure 7A, DC_50_ = 0.88 nM for compound **27**) or with a SuFEx‐based library of 3180 amines coupled to 5′‐amino‐lenalidomide (Figure 7B, compound **32**), tested across leukemia and neuroblastoma cell lines with HiBiT validation [75, 77]. Also, GSPT2 emerged as a hit in a newly established isogenic cell painting assay from a SuFEx screen involving six fluorosulfates (compound **28,** Figure 7B) and 21 aliphatic amines as a proof‐of‐concept [78]. In another study, over 20,000 CRBN‐based compounds were synthesized via amide formation and Buchwald–Hartwig coupling [76]. The resulting library was screened by ASMS against the lymphocyte‐specific tyrosine kinase (LCK), enabling targeted MG identification.

In contrast to these E3 ligase‐first approaches, some efforts have pursued a “degraded target”‐first strategy: For BRD4 and histone acylation reader eleven‐nineteen leukemia (ENL), known inhibitors (JQ1 and SR‐0813 (**30**), respectively) were functionalized with SuFEx‐compatible exit vectors and reacted with 3163 structurally diverse amines (Figure 7B) [73]. Screening via HiBiT yielded ENL degraders (e.g., compound **31**) with DC_50_ values from 50 nM to 1.85 µM, while CRISPR/Cas9‐mediated knockout of CRBN in MV4;11 cells confirmed that degradation was CRBN‐dependent.

### 3.5 Carbonic Anhydrase Inhibitors

Carbonic anhydrases (CAs) catalyze the hydration of CO_2_ to HCO_3_^−^, playing a central role in pH regulation [79]. Their inhibitors are utilized in diseases such as glaucoma, macular edema, and cancer [80]. Although carbonic anhydrase inhibitors are not new in the current focus of medicinal chemistry, there are several D2B development campaigns highlighting the model character of carbonic anhydrase for D2B development.

**FIGURE 8:**
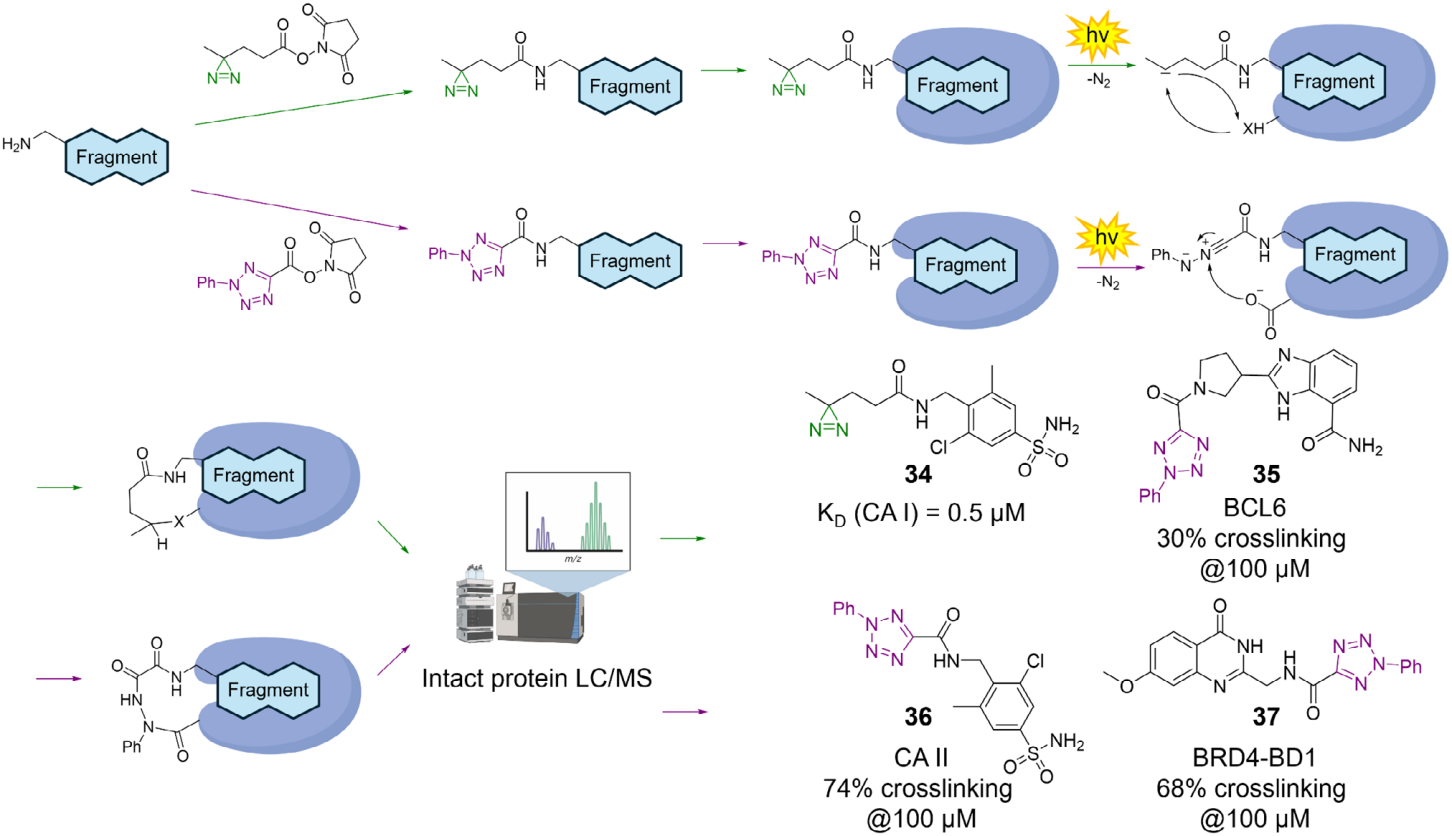
PhABit/D2B methodology introduced by Bush and coworkers. Scheme illustrating two different PhABits containing a diazirine chemotype (green) or 2‐aryl‐5‐carboxytetrazole chemotype (violet). Fragments containing amine groups are reacted with the respective photosensitive transfer reagent, which is then incubated with the target enzyme, irradiated, and analyzed by intact protein LC/MS. This methodology was used for D2B campaigns targeting CA I/II (**34**, **36**) and also BRD4‐BD1 (**35**, **37**) [1, 81].

To explore fragment–protein interactions beyond cysteine residues (as described for M^Pro^ above, Figure 3), new platforms for screening of covalent inhibitors were adapted by Bush and coworkers, targeting CAs, among others [1, 50, 81]. In their first strategy, a 1073‐membered alkyl‐amine fragment library was functionalized via NHS ester chemistry with a photoreactive alkyl diazirine moiety, enabling initial noncovalent binding followed by UV‐induced crosslinking, also dubbed the PhotoAffinity Bits (PhABits) methodology (Figure 8) [1, 82]. Displacement experiments with ethoxzolamide, a known inhibitor targeting the zinc‐binding site, confirmed active‐site engagement and yielded five primary sulfonamide‐containing hits, and subsequently, a focused library of 100 sulfonamide analogs was synthesized, resulting in several micromolar‐affinity binders (e.g., compound **34**) [1]. In a second strategy, photoactivatable 2‐aryl‐5‐carboxytetrazoles (ACT)‐PhABits targeting Asp/Glu residues were explored via amidation D2B and generated 17 hits for CA I and 16 for CA II (e.g., compound **36**) [81].

In a different CA‐targeting campaign, sulfur(VI) fluoride‐RFs targeting lysine, tyrosine, and serine yielded four initial hits; although initial *k*_inact_ values were low, an optimized 96‐member analog library achieved higher modification yields and improved selectivity (Figure 3, e.g., compound **8**) [50]. Collectively, these studies expand fragment‐based covalent ligand discovery beyond traditional cysteine targeting for CA I and CA II.

### 3.6 Antiviral and Antibacterial Drugs

The emergence of antibiotic resistance drives the search for new antibacterial agents as an ongoing priority in academic and industrial research [83]. Likewise, viral targets remain central to drug discovery efforts, particularly since the COVID‐19 pandemic [84]. It is therefore unsurprising that the D2B approach has been applied to both bacterial and viral targets beyond the SARS‐CoV‐2 main protease (chapter 3.2).

**FIGURE 9:**
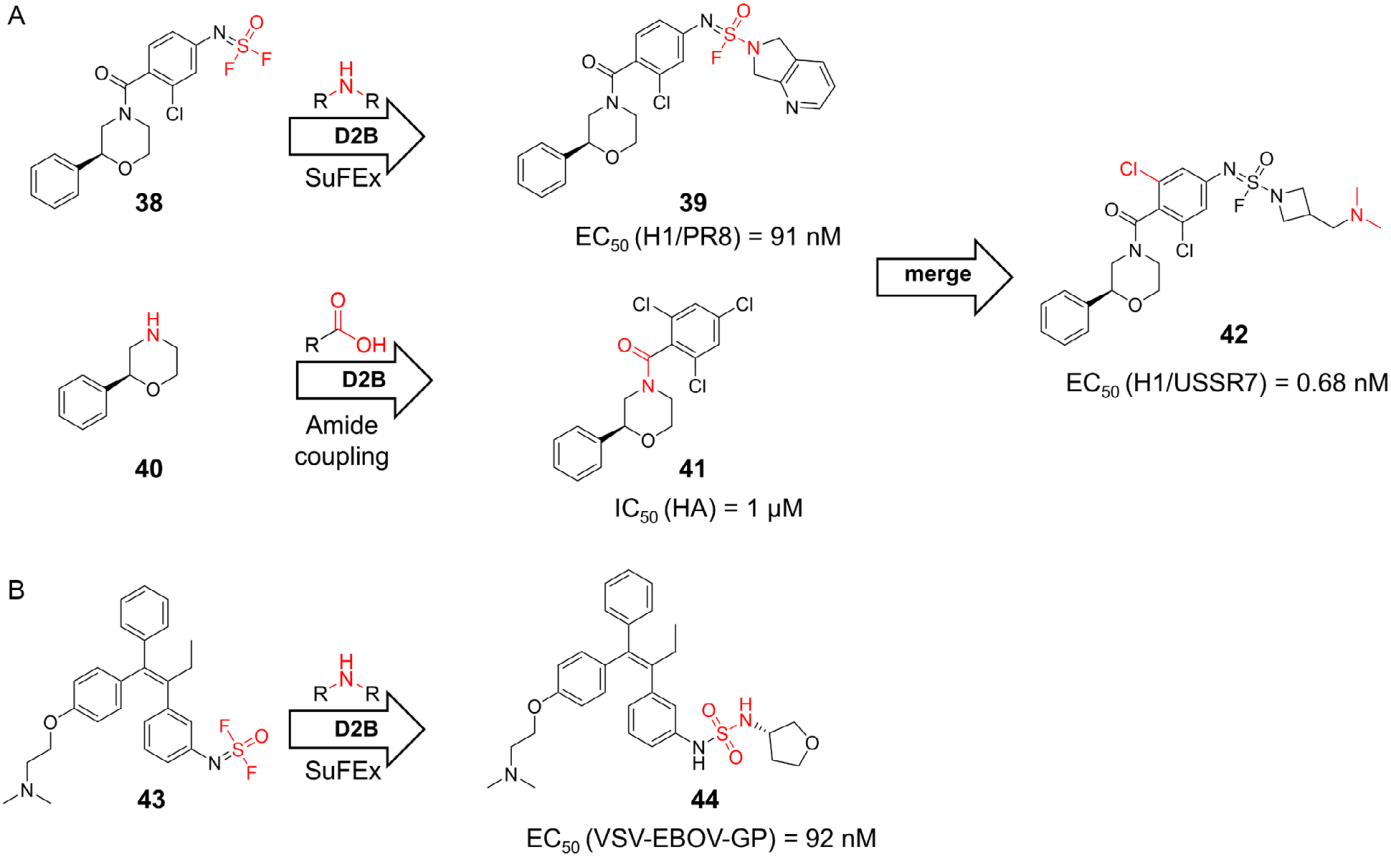
D2B campaigns targeting selected antiviral targets. (A) Scheme illustrating the workflow for the development of influenza virus HA inhibitors, combining SuFEx and amide coupling‐based D2B approaches to yield compound **42** [85]. (B) SuFEx‐based D2B strategy targeting EBOV GP, resulting in compound **44** [86].

Two prominent viral targets explored using D2B strategies are the influenza virus and Ebola virus (EBOV). In the case of influenza, the stem region of hemagglutinin (HA) was targeted by D2B‐derived inhibitors, and thus, building on a previously established high‐throughput FP assay, a fragment‐like ligand with an EC_50_ of 1.9 µM could be identified (**38**/**40**, Figure 9A) [87]. Subsequent X‐ray crystallography revealed three unoccupied regions, which were subsequently derivatized using SuFEx chemistry in a D2B manner [19, 87]. This enabled the generation of a 690‐membered library via reaction with 230 amines. In parallel, an amide library was synthesized by coupling an amine‐modified version of the fragment to 500 carboxylic acids. Both libraries were tested without purification in the FP assay, and the best compounds (**39** and **41**) from each library (SuFEx and amidation) were merged, yielding final compound **42** with sub‐nanomolar EC_50_ values across multiple H1 strains, representing the most potent HA stem binder reported to date [85].

For targeting EBOV, Kitamura and colleagues selected the EBOV glycoprotein (GP) as a potential D2B target [86]. In silico docking of tamoxifen to this receptor guided the strategy of derivatization, followed by SuFEx‐based modification using thionyl tetrafluoride to introduce a difluoride functionalization handle to yield compound **43**. A library of 2496 amines was subsequently coupled using an automated liquid handling robot, and the crude products were evaluated in a cell‐based EBOV entry assay. The top hit (**44**) of this study exhibited a *K*_D_ of 0.63 µM, an over 50‐fold improvement compared to tamoxifen (*K*_D_ = 32 µM), and an EC_50_ value of 92 nM in VSV‐EBOV‐GP (Vero cells) [86].

For D2B‐targeting of bacterial targets, several complementary approaches have been explored. In this regard, covalent fragment‐based drug discovery was applied to *Salmonella* and *Shigella* NEL E3 ligases by screening 227 electrophilic fragments bearing chloroacetamide warheads targeting cysteines (similar to the approach used for M^Pro^ and TRIM25 above, Figure 3); while no hits were found for IpaH (*Shigella*), three hits could be identified for SspH (*Salmonella*) [51]. A focused D2B library of 430 amines was used for hit expansion via amide coupling to chloroacetamide warheads, yielding compound **7** (Figure 3) that showed significant protein labeling at 6.25 µM and inhibition of PKN1 ubiquitination in a reconstituted ubiquitin enzymatic cascade [51].

Beyond cysteine‐targeting inhibition, Wennekes and coworkers investigated the dichlorotriazine (ClTriZ) covalent warhead for lysine targeting and optimized it using a biotin‐avidin model system [88]. Its integration into a modular “double‐click” (CuAAC and strain‐promoted azide–alkyne click chemistry reaction (SPAAC)) platform enabled sequential ligand and reporter diversification, yielding a panel of functionalized probes for *Photobacterium multocida* sialyltransferase 1 (PmST1) and supporting a D2B approach. This strategy was subsequently applied to generate and screen covalent ClTriZ‐based probes targeting *Neisseria gonorrhoeae* lipooligosaccharide sialyltransferase (Ngono Lst), enabling irreversible inhibition in complex lysates without purification [88]. In a separate D2B publication investigating nitrofuran‐based coupling products, 40 derivatives were tested in a nanoscale format, yielding potent MICs (0.8–7.8 µM) against *Staphylococcus aureus* and *Escherichia coli* [89]. Also, recently, a different antibacterial strategy for peptide nucleic acid (PNA)‐based antisense molecules targeting essential bacterial mRNAs, using 1536 sequences and truncation strategies [90].

One of the most recent studies in the D2B field targets DsbA, a bacterial oxidoreductase essential for the folding and function of virulence factors [91]. This enzyme features two key binding sites (a cryptic pocket and a hydrophobic loop). Starting from a fragment that bound to the cryptic pocket, a D2B strategy generated a library of amino alcohols containing a hydroxyethylamide moiety, crucial for engaging the interface of the cryptic pocket and hydrophobic loop. The crude library (92 members) was coupled to the phenylisoxazole core, which contains an alkyne linker with a carboxylic acid handle. The resulting products were screened using SPR and ASMS, and the most potent hit demonstrated a *K*_D_ of 1.5 µM (ITC).

A high‐throughput CuAAC‐based combinatorial platform was used to generate hundreds of transition‐metal complexes from a nanomole‐scale ligand library and to screen the crude libraries directly in biological assays. Using this D2B strategy, potent metalloantibiotics with nanomolar activity as well as catalytically active metal complexes were rapidly identified and validated by resynthesis [92].

Lastly, TrmD, an essential tRNA methyltransferase in various bacteria, was targeted using a D2B approach. Building on a previously identified pyrrolopyrimidine scaffold, an alkyne handle was introduced, and CuAAC was performed to link a diverse library of 320 azides. The compounds displayed IC_50_ and *K*_D_ values in the nano‐ to low‐micromolar range against *S. aureus* TrmD, with selectivity over TrmD from other species [93].

### 3.7 Other Targets and Inhibitors

Several additional D2B campaigns not categorized by the chapters above were conducted by Bush, Rittinger, and colleagues using RFs as previously discussed (Figures 3 and 8). The ACT‐PhABit approach (Figure 8) yielded 17 selective KRAS, 14 BCL6 hits (compound **35**), and BRD4‐BD1 (compound **37**), characterized by weak binding affinities, representing starting points for further optimization [81]. Sulfonyl fluorides, also applied in CA II targeting, were used to identify covalent RFs for BCL6, and thus, a focused library of 352 compounds, designed in a D2B manner, revealed meta‐substituted benzamides (compound **9**) as preferred covalent chemotypes (Figure 3) [50].

A similar strategy was applied for the development of covalent acrylamide‐based inhibitors of the Werner (WRN) helicase (Figure 3) [52]. Here, an initial LC/MS screen of 1055 acrylamides yielded two moderately affine hits (IC_50_ = 15.8 µM). Hence, two parallel optimization approaches were followed: one coupled 328 Tanimoto‐similar amines to the acrylamide electrophile, leading to a best hit with IC_50_ = 1 µM, the other employed multistep syntheses, including amide coupling and acrylamide formation, resulting in a compound with IC_50_ = 251 nM. Structural insights via X‐ray crystallography informed a third round of D2B optimization, using 123 amines to generate analogs, culminating in lead compound **10** with IC_50_ = 31.6 nM (Figure 3) [52].

Similarly, chloroacetamide‐based screenings to identify selective inhibitors of OTU domain‐containing protein 7B (OTUD7B), yielding a hit with IC_50_ = 3.5 µM [94]. In this study, targets labeled with fragments were identified via biotinylated ubiquitin vinyl sulfone probes and LC/MS from HEK lysates. Subsequently, a 351‐membered amine library was then coupled to a chloroacetamid electrophile and screened via intact protein LC/MS to yield the mentioned hit.

**FIGURE 10:**
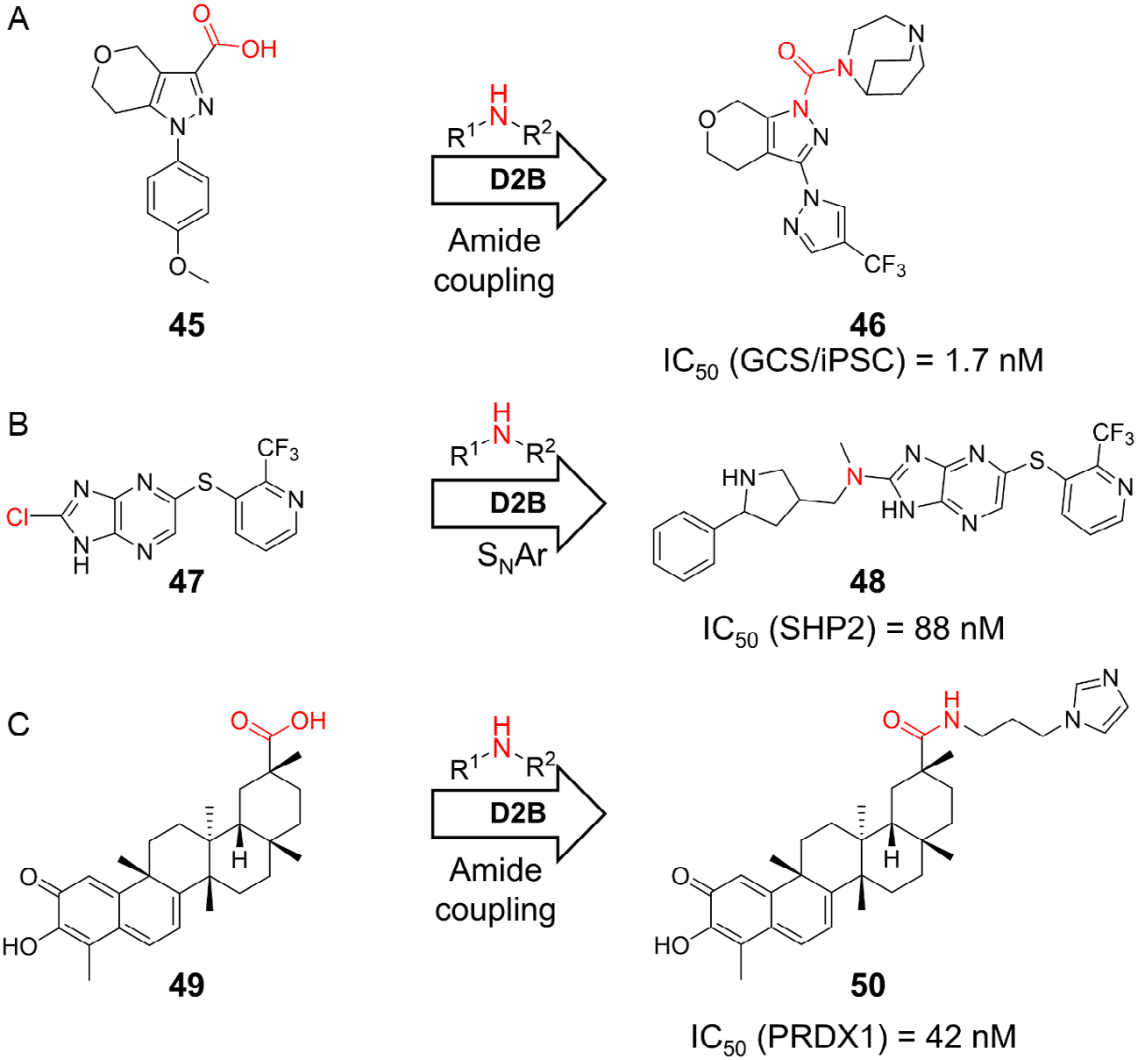
A selection of D2B campaigns targeting various other targets. (A) Amidation‐based D2B campaign by Roecker and coworkers targeting glucosylceramide synthase (GCS) for the treatment of Parkinson's disease [95]. (B) D2B campaign targeting SHP2, leading to the development of compound **48** [96]. (C) Identification of calosterol derivative **50** by Xiong et al. via an amidation‐based D2B campaign [97].

In another study, Roecker and coworkers, targeting glucosylceramide synthase (GCS), crucial in Parkinson's disease, identified a diazepane core (**45**) via HTS [95]. Guided by QSAR, they synthesized 1390 diazepane derivatives via HATU coupling and screened them directly in a GCS enzymatic assay, and following scaffold hopping, they identified compound **46** (Figure 10A) with picomolar potency in enzymatic assays and an IC_50_ of 1.7 nM in human iPSC‐derived neurons. Further, Ponzi et al. used SNAr chemistry to attach 160 amines to an imidazopyrazine scaffold (**47**) via automated synthesis, identifying Src homology region 2 tyrosine phosphatase 2 (SHP2) allosteric inhibitors with IC_50_ values of 225 nM and, after further improvement, 88 nM (compound **48**, Figure 10B) [96]. Lastly, Chen et al. generated 2720 celastrol derivatives targeting peroxiredoxins (PRDXs) [97]. The best compound **50** showed a 4.6‐fold improvement over celastrol in PRDX1 inhibition (IC_50_ = 42 nM) and selectivity over PRDX2/3/4/6 (Figure 10C).

## 4 Computational D2B Technologies

**FIGURE 11:**
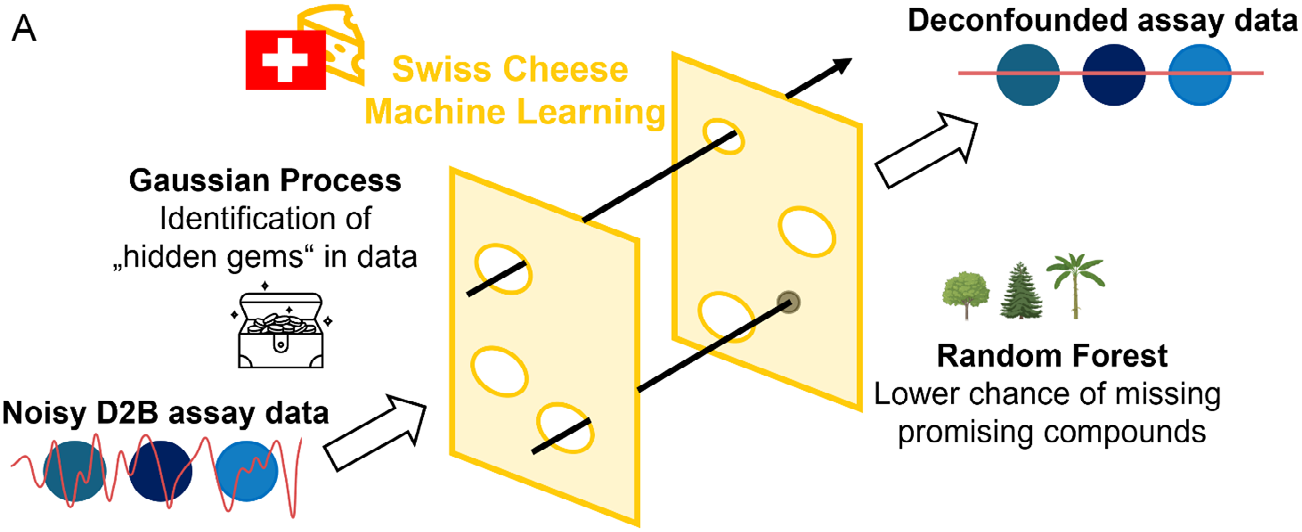
(A) The “Swiss Cheese” machine learning‐based deconfounder, based on Gaussian process and random forest models, to detect false negative results in noisy D2B assay data. Figure adapted from McCorkindale et al. [47].

Together with synthetic developments, computational methods have been leveraged to support D2B workflows and the analysis of HTS data. One such platform is the Cernak laboratory development “phactor”, a software tool designed to automate data handling and streamline reaction array setup and evaluation [45]. It processes diverse data types, including LC/MS and biochemical assay outputs, and is compatible with widely used instruments and programs by utilizing Excel and Python. Similarly, King‐Smith and coworkers employed an M^Pro^‐targeting campaign to validate a machine learning (ML)‐based “deconfounder” that distinguishes low potency from low synthetic yield, mitigating false negatives [47]. Their “Swiss cheese” method integrates random forest and Gaussian process models to identify consistent SAR patterns within noisy datasets and is extendable to in silico screening for novel scaffolds (Figure 11A).

In another publication, transfer learning of known antibiotics was applied to a chemical language model to find new antibacterial compounds de novo [89]. This enabled the generation of synthetically feasible, novel antibacterial structures. Subsequent D2B evaluation through amide coupling with 40 amines yielded compounds with promising MIC values against *S. aureus* and *E. coli*. Furthermore, the Biggin group analyzed data from a D2B crystallographic study by von Delft and Spencer using a so‐called “xSAR” approach. They extracted features from Morgan fingerprints and calculated positive and negative binding scores (PBS/NBS) based on conserved binding (CBB) and conserved nonbinding bits (CNB), ultimately identifying 26 previously classified as nonbinder PHIP2 binders [59, 60]. Computational tools developed specifically for D2B, such as phactor, are designed to operate on reaction‐ and plate‐native data structures, enabling the joint analysis of synthetic parameters and biological readouts in a manner not readily supported by conventional HTS software. Likewise, deconvolution frameworks such as the “Swiss cheese” model and xSAR leverage conserved reaction‐level or binding features to improve hit identification from noisy crude‐mixture data, providing capabilities that extend beyond traditional SAR modeling.

## 5 Limitations and Outlook of Direct‐to‐Biology

Despite unprecedented resource investment, the rate at which new drugs reach the market (or just enter clinical trials) has remained largely unchanged over the past 20 years [98]. To date, early drug discovery depends on time‐ and resource‐intensive synthetic workflows that are challenging to scale (especially in academia) and are often poorly integrated with their biological evaluation [99]. D2B workflows trade purification for speed and material economy, but that trade introduces specific and consequential limitations. The principal concern is matrix interference in biochemical and cell‐based assays [100, 101, 102, 103]. Reagents, side products, solvents, and residual catalysts can, in principle, interfere with biological assays, leading to false positives (assay readout driven by an impurity rather than the intended compound) or false negatives (loss of signal due to inhibitory or quenching components). A corollary is assay dependence; fluorescence‐ and luminescence‐based readouts are particularly susceptible to quenching or background signal from reaction components, whereas label‐free biophysical methods (e.g., mass spectrometry) are generally less prone to such artifacts. Assay interference in D2B campaigns most commonly arises from residual reaction components, including trace transition metals (e.g., Pd from cross‐coupling reactions or Cu from CuAAC) and reactive reagents that can directly modulate protein activity or perturb assay readouts in a concentration‐dependent manner [104]. To mitigate such effects, several studies recommend matrix‐spiking controls, metal scavengers, and orthogonal assay formats to distinguish true compound activity from artefactual signals originating from the crude reaction mixture. To mitigate matrix‐dependent assay interference, some recent studies recommend project‐agnostic D2B compatibility tests, in which crude but structurally irrelevant reactions are spiked with positive controls to probe assay robustness. Nonetheless, such procedures add overhead and may reveal that certain reaction types or assay formats will be simply unsuitable for D2B.

On the chemistry side, D2B currently favors a constrained subset of transformations that are robust, high‐yielding, and assay‐tolerant. This selection of chemistry aligns well with the needs of academic and early pharma medicinal chemistry campaigns. However, when comparing the frequency of chemotypes found in approved drugs with the current D2B toolbox, there is a strong need to expand the reaction repertoire to the benefit of C—C/C—N cross‐couplings and heterocyclization reactions. Across reported campaigns, reaction success rates are not uniformly defined or disclosed; however, where such metrics are available, click‐chemistry‐based transformations (e.g., CuAAC, SuFEx, and amide coupling) consistently achieve success rates exceeding 90%. In contrast, more complex transformations such as Pd‐catalyzed cross‐couplings often display markedly lower success rates (mostly <50%), revealing a pronounced practical divide between chemistries that are readily compatible with D2B formats and those that remain challenging under miniaturized, purification‐free conditions. Furthermore, attempts to push more sensitive or complex chemistries to nanomole, plate‐format conditions expose issues of incomplete conversion, reagent decomposition, and poor mixing (diffusion limits in 384/1536 wells), which can impair hit identification or translatability to batch synthesis [105].

Analytical throughput and decision‐making are further bottlenecks: mass spectrometric profiling and data parsing for hundreds of crude wells are time‐consuming and require semiautomated pipelines and bespoke thresholds for acceptable conversion, thresholds that depend on both assay sensitivity and project context. Finally, because only resynthesized hits are validated, early SAR can be clouded by artifacts in the crude matrix, so D2B is currently best deployed as a filtering strategy that demands rigorous controls, orthogonal confirmatory assays, and conservative interpretation until purified follow‐up data are available.

Together, these considerations underscore that D2B is not yet a universal replacement for conventional synthesis‐purification‐assay pipelines. It is best viewed as a complementary strategy that, when applied with rigorous controls and awareness of its limitations, can substantially accelerate the early discovery cycle while avoiding costly conventional SAR dead ends.

Looking forward, D2B is expected to expand both in methodological breadth and translational scope. Future expansion of D2B chemistry will likely depend on adapting additional reaction classes to the stringent constraints of miniaturized, purification‐free workflows. While transformations such as photoredox catalysis or late‐stage C—H functionalization are conceptually attractive due to their ability to access new chemical space, their implementation in true D2B formats remains largely unexplored and will require careful validation. Addressing challenges related to reaction control, reproducibility, and assay compatibility will be essential before such chemistries can be reliably integrated into D2B campaigns.

Increasing engagement from commercial providers (for D2B service and for affordable building block libraries) underscores that D2B is moving from proof‐of‐concept toward a standardized discovery tool. Ultimately, the field will need to demonstrate consistent success across diverse targets and chemistries to solidify its role as a mainstream complement to traditional library‐based discovery. To this end, further development and more frequent use of computer‐assisted methods for designing and evaluating D2B campaigns will certainly play a major role in the near future.

## Funding

Open Access funding enabled and organized by Projekt DEAL.

## Conflicts of Interest

The authors declare no conflicts of interest.
